# Injury specific trauma registry: Outcomes of a prospective cohort with proximal femur fractures

**DOI:** 10.1016/j.amsu.2019.07.015

**Published:** 2019-07-11

**Authors:** Tashfeen Ahmad, Zehra Abdul Muhammad, Ahmed Habib

**Affiliations:** a Departments of Surgery and Biological & Biomedical Sciences, Aga Khan University, Karachi, Pakistan; b Department of Surgery, Aga Khan University, Pakistan

**Keywords:** Trauma registry, Neck of femur fractures, Intertrochanteric fractures, Outcomes

## Abstract

**Background:**

The elderly population is prone to hip fractures, and treating such patients to achieve good outcomes can be challenging. Collection of outcomes data can support clinicians to modify their treatment protocols and improve outcomes over time. The aim of this study is to compare different surgical procedures in patients with neck of femur and intertrochanteric fractures in terms of clinical, functional and radiological outcomes using injury-specific outcome scores.

**Methods:**

The study data was derived from the existing single-center, prospective orthopaedic trauma registry initiated from July 2015. Functional, clinical and radiological outcomes were assessed using Modified Harris Hip Score and The Radiographic Union Score for Hip. Mean radiological outcome scores was compared by Mann-Whitney U test and deaths by Chi-square and Odds ratio.

**Results:**

Of the total 138 patients, 53 (38%) were neck of femur and 85 (62%) Intertrochanteric fractures with fall as leading cause of injury. At 12 months follow-up, modified Harris Hip Score showed 67% excellent-good results in both dynamic hip screw (N = 6) and total hip replacement (N = 3) followed by 50% in intramedullary nail (N = 2). Hemiarthroplasty has fair-poor outcomes with significantly higher deaths as compared to other procedure groups (*p* = 0.016). Radiological outcomes showed non-significant trend towards better outcomes in dynamic hip screw as compared to intramedullary nail (*p* = 0.08).

**Conclusion:**

Our 12 months follow-up data suggest that dynamic hip screw and total hip replacement have better clinical, functional outcomes followed by intramedullary nail. Hemiarthroplasty has fair-poor clinical and functional outcomes with significantly higher deaths as compared to other procedure groups.

## Highlights

• To compare different surgical procedures in patients with neck of femur and intertrochanteric fractures in terms of outcomes.
• A Single center, prospective cohort study.
• Dynamic hip screw, total hip replacement, intramedullary nail, hemiarthroplasty.
• Functional, clinical and radiological outcomes assessment using Modified Harris Hip Score and The Radiographic Union Score.
• At 12 months, DHS and THR have better outcomes. Hemiarthroplasty has significantly higher deaths as compared to other groups.

## 1 Introduction

Patients’ sustaining proximal femur fractures pose a major challenge to the health care team due to the old age that majority of such patients belong to, related risk factors, prolonged period of recovery and high mortality rate [1,2]. Ninety five percent of proximal femur fractures in elderly patients is due to fall [3]. There is a high risk of mortality after proximal femur fractures particularly in elderly patients 60 years of age or above. According to one study, overall 1-year mortality in patients ≥60 years of age treated after proximal femur fractures was 21%. In another research overall 1-year postoperative mortality was 27% [4,5]. Depending on the fracture type and clinicians preference, different surgical procedures are chosen to manage these fractures [6], in order to achieve the best outcomes considering the fracture and patient characteristics.

Validated scoring scales provide valuable information about the patients’ progress after proximal femur fracture management, and can show which procedure has the best outcome [7,8]. Modified Harris Hip Score (modified HHS) has acceptable validity to assess functional and clinical outcomes after proximal femur fracture management [9,10]. To assess radiological outcomes, obliteration of fracture line and cortical bridging through callus formation are valid indicators of bone healing. The Radiographic Union Score for Hip (RUSH) score (maximum 30 points) is designed to evaluate proximal femur fracture healing after treatment [11]. We hypothesized that improvement in clinical, functional and radiological outcomes of proximal femur fractures treated with different surgical approaches can be objectively assessed using modified HHS and RUSH scoring scales, as these are applicable to proximal femur fractures and thus injury-specific. Therefore, we aim to compare clinical, functional and radiological outcome scores of patients sustaining neck of femur (NOF) and intertrochanteric (IT) fractures at defined follow-up visits treated with different surgical procedures.

## 2 Methods

A prospective cohort study was initiated in 2015 in which cases of upper and lower limb trauma were recruited for an orthopaedic trauma registry. The registry captures injury specific data on injury patterns, causes of injury, demography, injury management as well as functional, clinical and radiological outcomes at defined follow-up visits. Institutional and Ethical Review Committee approvals (reference number 0525–540) were obtained. The study was registered at Research Registry with UIN number researchregistry3466 for public access and the study protocol can be requested from corresponding author. The current analysis presented in this paper is based on registry data of isolated traumatic neck of femur (NOF) and intertrochanteric (IT) fractures in patients presenting between July 2015 and July 2018. Irrespective of age and gender, all eligible patients, seeking care at Aga Khan University Hospital with isolated traumatic NOF and IT fractures were included. Patients with pathological fractures and multiple fractures were excluded. Written informed consent was obtained prior to data collection. Trauma related data was obtained from patient's medical record and their outcomes were assessed by experienced research associate at 3 months ± 2 weeks, 6 ± 1 months and 12 ± 2 months after treatment. A total of 138 patients with isolated proximal femur fracture were recruited, comprising 53(38.4%) femur neck and 85(61.6%) intertrochanteric fractures. Post-treatment functional and clinical outcomes were assessed using modified HHS categorized as < 70, poor; 70–79, fair; 80–89, good; and 90–100 excellent. Radiographic healing of fractures treated with dynamic hip screw (DHS) or intramedullary nail (IM nail) was assessed by RUSH score. RUSH score was not applicable for patients undergoing total hip replacement (THR) and hemiarthroplasty. Data was analyzed on Statistical Package for the Social Sciences (SPSS) version 19.0 and was cross checked by the principal investigator. Descriptive analysis was performed for age, gender, mechanism of injury and type of management (surgical/non-surgical). Continuous variables were expressed as mean ± standard deviation and categorical variables as percentages (%). The *p*-value of less than 0.05 was considered as statistically significant with a confidence interval of 95%. Non-parametric Mann-Whitney U test was performed to compare significant difference between radiological outcomes of DHS and IM nail. Association of DHS or IM nail with mean RUSH score (maximum 30 and minimum 10) was assessed at 3 months ± 2 weeks, 6 ± 1 months and 12 ± 2 months follow-up visits to assess radiological bone healing. Deaths from the time of surgery to 12 months were analyzed by Chi-square test and Odds ratio (OR).

## 3 Results

**Fig. 1:**
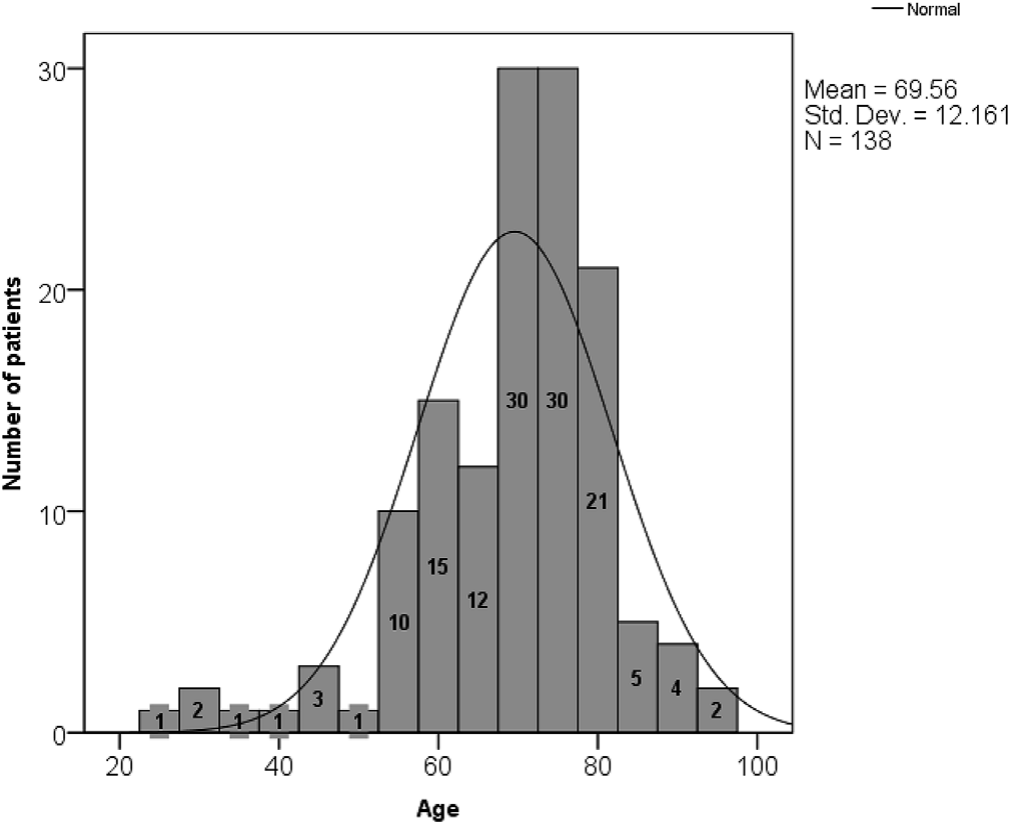
Patients age distribution.

**Fig. 2:**
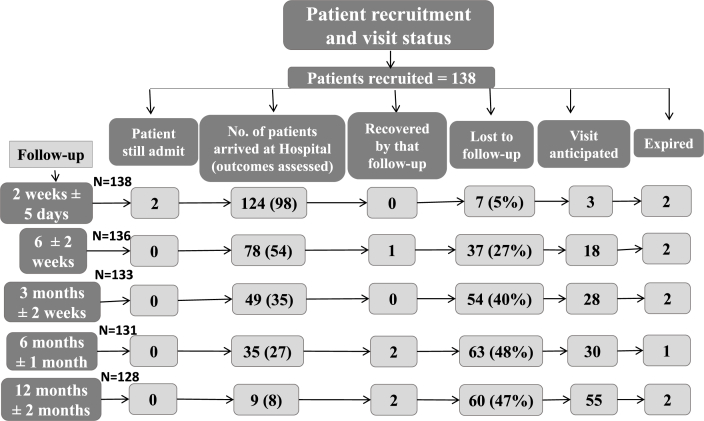
Patient Recruitment and visit status.

**Fig. 3:**
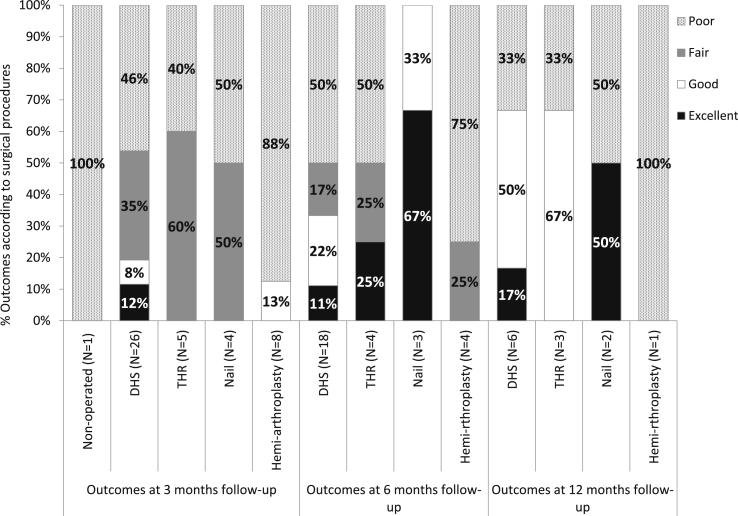
Clinical and Functional outcomes at Follow-up According to Surgical Procedure.

A total of 138 patients with NOF (N = 53) and IT (N = 85) fractures were recruited in which 80 (58%) were females and 58 (42%) were males. Mechanism of injury was fall in 127 (92%) followed by RTA in 9 (6.5%) and assault in 2 (1.5%) patients. Ninety seven percent (134 patients) were operated who underwent different surgical procedures including Dynamic hip screw (DHS, N = 79), hemiarthoplasty (N = 31), total hip replacement (THR, N = 18) and IM nail (N = 6) (Table 1). Twenty two patients were under 60 years of age (16%) while 116 (84%) were 60 years or more (Fig. 1). Status of patient recruitment and visits was recorded (Fig. 2). At 6 months follow-up, modified HHS Score with mean ± SD was 88.7 ± 5.6 for IM nail (N = 3), 71.7 ± 13.8 for DHS (N = 18), 56.9 ± 32.3 for THR (N = 4) and 54.2 ± 23.5 for hemiarthoplasty (N = 4) demonstrating 100% excellent-good results in IM nail followed by 33% in DHS and 25% in THR. At 12 months follow-up visit, excellent-good results were 67% in both DHS (N = 6) and THR (N = 3) followed by 50% in IM nail (N = 2) (Fig. 3).

**Table 1:** Proximal femur fractures Characteristics.

Characteristics of Fractures		
Type of Fracture	Neck of Femur	53 (38%)
	Intertrochanteric	85 (61.6%)

Mechanism of injury	Fall	127 (92%)
	RTA	9 (6.5%)
	Assault	2 (1.5%)

Operated vs non-operated	Operated	134 (97%)
	Non-operated	4 (3%)

Procedures	Dynamic Hip Screw	79 (57%)
	Intramedullary nail	6 (4%)
	Hemiarthroplasty	31 (22.5%)
	Total Hip Replacement	18 (13%)
	Conservative	4 (3%)

Total nine patients expired of which 5 (55.5%) had IT and 4 (44.5%) had NOF fracture. Five patients expired after hemiarthroplasty, 3 after DHS and 1 patient was non-operated due to severe comorbid condition. Five out of nine deaths were within 2 months post procedure in which 1 expired after DHS due to sepsis, 4 expired after hemiarthroplasty and reason was sepsis in 3 patients and 1 was dead on arrival thus, possibly suggesting surgical related cause. The rest of 4 patients expired due to non-surgical reasons like urosepsis, aspiration pneumonia, dead on arrival after 9 months post-procedure and sepsis with diabetes mellitus (DM) and chronic kidney disease (Table 2). Using Chi-square test and OR from the time of surgery to 12 months post procedure, comparison was applied to DHS versus hemiarthroplasty group, hemiarthroplasty versus the rest of procedure groups and hemiarthroplasty versus combined THR and IM nail groups. Difference in deaths between DHS versus hemiarthroplasty group was significant (*p* = 0.039); odds of death in hemiarthroplasty group were 4.8 times those in DHS group. There was also significant difference in deaths between combined groups of DHS, THR and IM nail versus hemiarthroplasty (*p* = 0.016) with OR 6.4 for death in hemiarthroplasty compared to the other groups combined. Difference in deaths between hemiarthroplasty versus combined groups of THR and IM nail was significant (*p* = 0.049) (Table 2).

**Table 2:** Deaths (from the time of surgery to 12 months).

Surgical Procedures	Number of deaths (%)
Dynamic Hip Screw	3 (33%) 2 months post-procedure: 1 death *(sepsis)* After 5 and 7 months: 2 deaths *(aspiration pneumonia and urosepsis)*
Hemiarthroplasty	5 (55.5%) 2 months post-procedure: 4 deaths *(3 sepsis and 1 dead on arrival)* After 5 and 7 months: 1 death *(Sepsis due to DM and chronic kidney disease)*
Non-operated case	1 (11%) *(Dead on arrival after* 9 months *post-procedure)*
Intramedullary nail	0
Total Hip Replacement	0
**Surgical Procedure groups**	***p*-value and OR**
Dynamic Hip Screw versus hemiarthroplasty group	*p* = 0.039, OR = 4.8
Dynamic Hip Screw, Total Hip Replacement and IM nail groups versus hemiarthroplasty group	*p* = 0.016, OR = 6.4

OR = Odds Ratio, *p* = *p*-value for the difference in deaths between surgical procedures.

For radiological outcomes, mean RUSH score was 22 ± 5.5 for DHS (N = 25) and 20 ± 5.5 for IM nail (N = 4) at 3 months follow-up, 24.8 ± 5.4 for DHS (N = 23) and 25 ± 7 for IM nail (N = 2) at 6 months and 28.6 ± 2.4 for DHS (N = 6) and 26 for IM nail (N = 1) at 12 months follow-up. At 3, 6 and 12 months follow-up visits, Mann-Whitney U test presented non-significant difference in bone healing between DHS and IM nail groups.

## 4 Discussion

In our study, the most common mechanism of IT and NOF fractures was fall (92%) supporting previously published studies with increasing risk in elderly at age of 60 years or more [12].

Current research showed patients with IT and NOF fractures who underwent DHS and THR procedures have 67% excellent to good clinical and functional outcomes at 12 months follow-up followed by IM nail (50%) group. Mean radiological outcome scores were also higher in DHS treated group as compared to IM nail group. RUSH score was not applicable for hemiarthroplasty and THR groups therefore not included in radiological outcomes analysis.

There were significantly (*p* = 0.016) higher deaths in hemiarthroplasty group (16%) as compared to other procedure groups (3%). Three patients expired in DHS procedure group with one patient developed post-surgical sepsis while two expired due to aspiration pneumonia and urosepsis representing non-surgical reason. Statistically significant higher mortality in hemiarthroplasty group was observed as compared to DHS group (*p* = 0.039). Expiries in non-operated cases were excluded in analysis due to severe comorbid conditions. Although there was no expiry in IM nail and THR groups, sample size was much smaller than DHS group thus we cannot compare the outcome.

Hemiarthroplasty has fair-poor outcomes at 6 as well as 12 months follow-up visits. No specific surgery associated reason identified for fair-poor outcomes except for one patient with implant displacement but patients were aged between 68 and 79 years having serious comorbid conditions like cancer, IHD, hypothyroidism, DM and osteoporosis. Overall mortality was higher in hemiarthroplasty group as compared to the rest of the treatment approaches. Further evaluation elucidated that except for two patients, the main reason of death was sepsis within 6 weeks post procedure while one was dead on arrival and one expired due to infection after DM and chronic kidney disease.

Considering all the results, our study suggests that in patients with IT and NOF fractures, DHS and THR have the best outcome. Number of patients in the IM nail group was small, and larger sample size is required to determine for reliable information on that group.

## 5 Strength

1. Follow-up data was obtained directly from patients at follow-up visits, which is more reliable than telephonic interviews.
2. The use of validated instruments permitted determination of association between care provided and the outcomes.

## 6 Limitations

1. Small sample size of IM nail and THR groups, inadequate power of study to detect significant differences in outcome measures in these groups.
2. Clinical variables like patient comorbid conditions may have affected outcomes.
3. Unscheduled of follow-up visits and insufficient human resources for outcome assessment in all patients whenever they presented.

## Ethical approval

Institutional and Ethical Review Committee approvals (reference number 0525–540) were obtained by the Aga Khan University Hospital.

## Sources of funding

The study was supported by the Aga Khan University Hospital, Department of Surgery.

## Author contribution

**Dr. Tashfeen Ahmad:** The principal investigator contributed in study design, data analysis, and critical review of the manuscript.

**Dr. Zehra Abdul Muhammad:** Contributed in data collection, data management, data analysis, clinical and functional outcomes assessment and manuscript writing.

**Dr. Ahmed Habib:** Contributed in radiological outcomes assessment and manuscript review.

## Conflicts of interest

We have no conflict of interest to declare.

We certify that we have no affiliation with or financial involvement with any organization or entity with financial or other interest in the matter discussed in the manuscript.

## Research registration number

Research Registry with UIN number researchregistry3466.

## Guarantor

All three authors listed take full responsibility as guarantor.

## Provenance and peer review

Not commissioned, internally peer reviewed.
